# Supplementary material for: Content-rich biological network constructed by mining PubMed abstracts
Source: BMC Bioinformatics. 2004 Oct 8;5:147. doi: 10.1186/1471-2105-5-147 (PMC528731; doi:10.1186/1471-2105-5-147)
Supplement: Additional File 2 — The original results of the above study (non-essential files are deleted to keep the file size under the limit set by BMC bioinformatics). [file 1471-2105-5-147-S2.bz2 › chilibotAdditionalFile2/dip10/537ID10611280E10886/html/AR_NR2C2.html]

 


 **AR** and **NR2C2** 
  
Found 11 abstracts in PubMed, retrieved 10.  
 

 What does Google say? 
 PDF only 
| .edu only 

---

**Interactive relationship** (e.g. stimulation, inhibition, etc)

**Inhibitory relationship**- Here we demonstrate that the  **AR**  can interact with the testicular orphan receptor 4 TR4  [ **NR2C2** ]  and function as a repressor to down regulate the TR4  [ **NR2C2** ]  target genes by preventing the TR4  [ **NR2C2** ]  binding to its target DNA.  Ref: 10611280 Proc Natl Acad Sci U S A, 1999
**Neutral relationship**- Interestingly, the heterodimerization of  **AR**  and TR4  [ **NR2C2** ]  also allows TR4  [ **NR2C2** ]  to repress  **AR**  target gene expression.  Ref: 10611280 Proc Natl Acad Sci U S A, 1999
- Our aim was to pharmacologically characterize the effect of a nonpeptide NK1 receptor antagonist TAK  [ **NR2C2** ]  637  **aR** , 9R 7 bis trifluoromethyl benzyl, 1 1 tetrahydro 9 methyl 5 4 methylphenyl 7H diazocino g naphthyridine 3 dione and determine key mechanisms of TAK  [ **NR2C2** ]  637 action in the gastrointestinal tract.  Ref: 11861814 J Pharmacol Exp Ther, 2002

**Non-interactive relationship** (e.g. studied together, co-existance, homology, etc.)

- TAK  [ **NR2C2** ]  637  **aR** , 9R 7 bis trifluoromethyl benzyl, 1 1 tetrahydro 9 methyl 5 4 methylphenyl 7H diazocino g naphthyridine 3 dione is a novel tachykinin NK 1 receptor antagonist that has been shown to inhibit the micturition reflex in guinea pigs.  Ref: 10924932 Eur J Pharmacol, 2000
- Together, these data demonstrate that the coupling of two different receptors, through the heterodimerization of  **AR**  and TR4  [ **NR2C2** ] , is a unique signaling pathway in the steroid receptor superfamily, which may facilitate further understanding of the complicated androgen action in prostate cancer or libido.  Ref: 10611280 Proc Natl Acad Sci U S A, 1999
- In this study, we investigated the role of tachykinin NK1 receptors in the visceral pain response abdominal muscle contraction caused by colorectal distention in rabbits previously subjected to colonic irritation, using the selective tachykinin NK1 receptor antagonists TAK  [ **NR2C2** ]  637  **aR** , 9R 7 Bis trifluoromethyl benzyl, 1 1 tetrahydro 9 methyl 5 4 methylphenyl 7H diazocino g naphthyridine 3 dione and CP 9 94 2S, 3S 3 2 methoxybenzylamino 2 phenylpiperidine.  Ref: 11861799 J Pharmacol Exp Ther, 2002
- The suppression of TR4  [ **NR2C2** ]  mediated transactivation is selective because TRA16 shows only a slight influence on the transactivation of androgen receptor  [ **AR** ] , glucocorticoid receptor, and progesterone receptor.  Ref: 12486131 J Biol Chem, 2003
- The effect of TAK  [ **NR2C2** ]  637  **aR** , 9R 7 bis trifluoromethyl benzyl, 1 1 tetrahydro 9 methyl 5 4 methylphenyl 7H diazocino g naphthyridine 3 dione, a tachykinin NK1 receptor antagonist, on lower urinary tract function was investigated in cats.  Ref: 11459118 Jpn J Pharmacol, 2001
- Furthermore, by using a chimeric receptor beta 2  **AR**  Rfz 2 containing the ligand binding and transmembrane segments from the beta 2 adrenergic receptor beta 2  **AR**  and the cytoplasmic domains from rat Frizzled 2 Rfz 2, stimulation with the beta adrenergic agonist isoproterenol activates activities of endogenous CaMKII, TAK1  [ **NR2C2** ] , and NLK and inhibits beta catenin induced transcriptional activation.  Ref: 12482967 Mol Cell Biol, 2003
- The effects of a new tachykinin NK 1 receptor antagonist,  **aR** , 9R 7 bis trifluoromethyl benzyl, 1 1 tetrahydro 9 methyl 5 4 methylphenyl 7H diazocino g naphthyridine 3 dione TAK  [ **NR2C2** ]  637, on the micturition reflex were compared with those of drugs used for abnormally frequent micturition or incontinence.  Ref: 10812055 Eur J Pharmacol, 2000
